# Supplementary material for: The Construction and Exploration of a Comprehensive MicroRNA Centered Regulatory Network in Foxtail Millet (Setaria italica L.)
Source: Front Plant Sci. 2022 May 6;13:848474. doi: 10.3389/fpls.2022.848474 (PMC9121102; doi:10.3389/fpls.2022.848474)
Supplement: Supplementary file 1 [file Data_Sheet_1.zip › Sit-miR396a_Seita.4G166300.1_2457_TPlot.pdf]

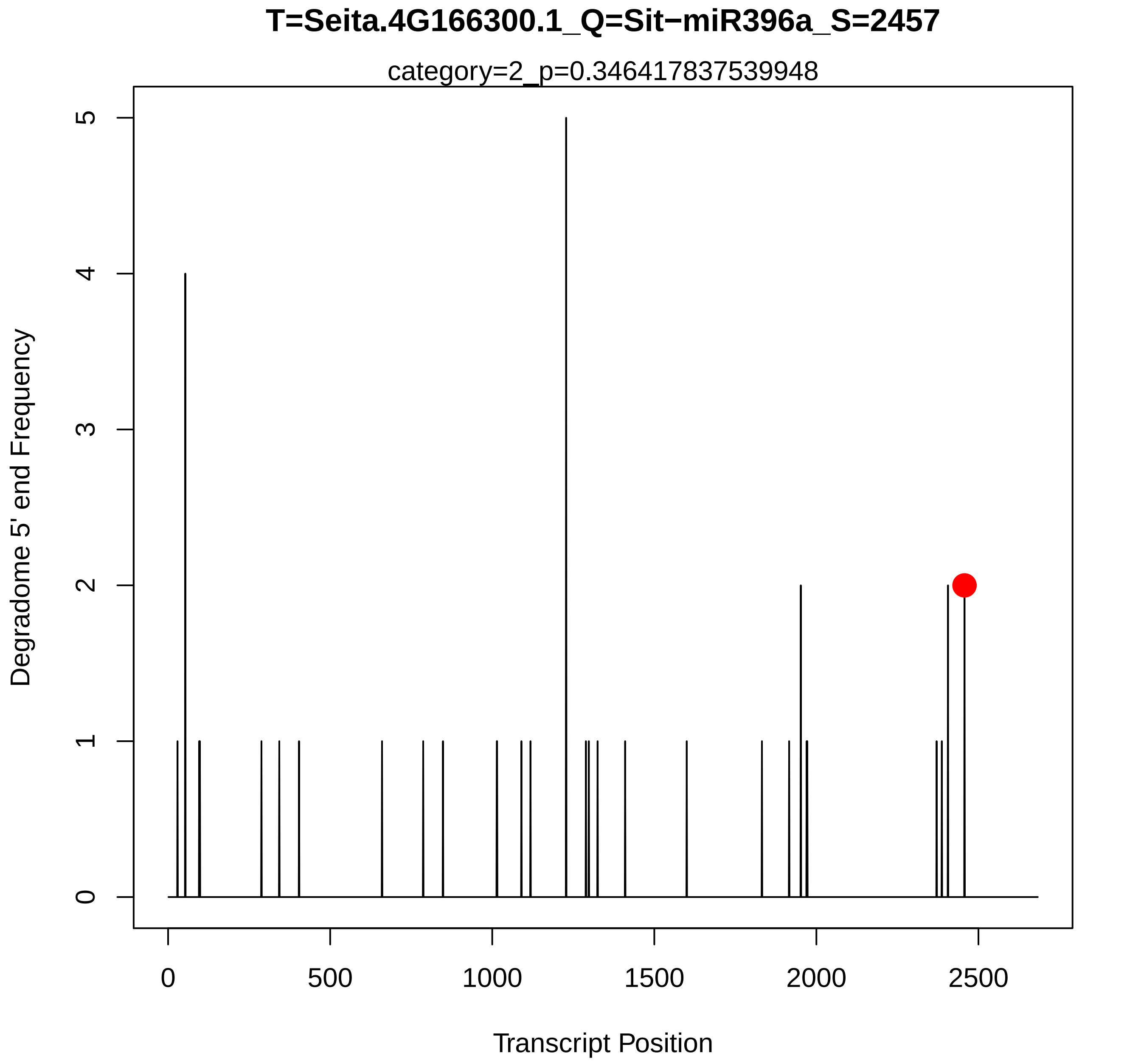

T=Seita.4G166300.1_Q=Sit−miR396a_S=2457
category=2_p=0.346417837539948
Degradome 5' end Frequency
Transcript Position
0
1
2
3
4
5
0
500
1000
1500
2000
2500
